# Supplementary material for: The impact of loneliness on depression, mental health, and physical well-being
Source: PLoS One. 2025 Jul 9;20(7):e0319311. doi: 10.1371/journal.pone.0319311 (PMC12240311; doi:10.1371/journal.pone.0319311)
Supplement: S1 Table — (DOCX) [file pone.0319311.s001.docx]

Supplementary Table S1: Racial and Ethnic Differences in the Association Between Loneliness and Depression (Marginal Effects Model)

|  | Race & Ethnicity | Margin | Std. Err. | t | 95% CI | | P>t |
| --- | --- | --- | --- | --- | --- | --- | --- |
| **Lonely** | **Depression** |  |  |  |  |  |  |
| Never | Black vs. White | -0.095 | 0.028 | -3.39 | -0.15 | -0.041 | **0.001** |
|  | Hispanic vs. White | -0.096 | 0.037 | -2.57 | -0.169 | -0.023 | **0.01** |
| Always | Black vs. White | -0.256 | 0.069 | -3.71 | -0.391 | -0.121 | **<0.001** |
|  | Hispanic vs. White | -0.143 | 0.062 | -2.27 | -0.266 | -0.019 | **0.023** |
| Usually | Black vs. White | -0.173 | 0.067 | -2.59 | -0.304 | -0.042 | **0.01** |
|  | Hispanic vs. White | -0.12 | 0.06 | -1.98 | -0.239 | -0.001 | **0.048** |
| Sometimes | Black vs. White | -0.107 | 0.033 | -3.27 | -0.17 | -0.043 | **0.001** |
|  | Hispanic vs. White | -0.11 | 0.029 | -3.82 | -0.166 | -0.054 | **<0.001** |
| Rarely | Black vs. White | -0.091 | 0.026 | -3.53 | -0.141 | -0.04 | **<0.001** |
|  | Hispanic vs. White | -0.069 | 0.034 | -2.03 | -0.135 | -0.002 | **0.042** |

*Table 3 presents the marginal effects comparing Black and Hispanic individuals to White individuals in the association between loneliness and depression. Estimates reflect the difference in predicted probability of depression across loneliness categories by race and ethnicity. Models were adjusted for age, sex, marital status, employment, education, language, metro status, and included state, year, and month fixed effects. Negative values indicate a lower predicted probability of depression relative to White individuals. All comparisons are statistically significant at p < 0.05.*
