# Supplementary material for: The impact of loneliness on depression, mental health, and physical well-being
Source: PLoS One. 2025 Jul 9;20(7):e0319311. doi: 10.1371/journal.pone.0319311 (PMC12240311; doi:10.1371/journal.pone.0319311)
Supplement: S2 Table — (DOCX) [file pone.0319311.s002.docx]

Supplementary Table S2: Age Differences in the Association Between Loneliness and Depression (Marginal Effects Model)

|  | Age group | Margin | Std. Err. | t | 95% CI |  | P>t |
| --- | --- | --- | --- | --- | --- | --- | --- |
| Lonely | Depression |  |  |  |  |  |  |
| Never | 45-64Yr. vs. 18-44Yr. | 0.01 | 0.025 | 0.39 | -0.039 | 0.058 | 0.697 |
|  | >64Yr. vs. 18-44Yr. | -0.075 | 0.026 | -2.82 | -0.126 | -0.023 | **0.005** |
| Always | 45-64Yr. vs. 18-44Yr. | 0.049 | 0.042 | 1.16 | -0.034 | 0.132 | 0.247 |
|  | >64Yr. vs. 18-44Yr. | -0.111 | 0.057 | -1.92 | -0.223 | 0.002 | 0.055 |
| Usually | 45-64Yr. vs. 18-44Yr. | -0.037 | 0.037 | -0.93 | -0.114 | 0.041 | 0.352 |
|  | >64Yr. vs. 18-44Yr. | -0.057 | 0.054 | -1.06 | -0.162 | 0.048 | 0.29 |
| Sometimes | 45-64Yr. vs. 18-44Yr. | -0.036 | 0.021 | -1.69 | -0.078 | 0.006 | 0.092 |
|  | >64Yr. vs. 18-44Yr. | -0.127 | 0.026 | -4.84 | -0.178 | -0.076 | **<0.001** |
| Rarely | 45-64Yr. vs. 18-44Yr. | 0.002 | 0.019 | 0.13 | -0.034 | 0.039 | 0.898 |
|  | >64Yr. vs. 18-44Yr. | -0.051 | 0.024 | -2.12 | -0.1 | -0.004 | **0.034** |

*Table 4 presents the marginal effects comparing middle-aged (45–64 years) and older adults (>64 years) to younger adults (18–44 years) in the association between loneliness and depression. Estimates represent differences in predicted probability of depression across loneliness categories by age group. Models were adjusted for race/ethnicity, sex, marital status, employment, education, language, metro status, and included state, year, and month fixed effects. Negative margins indicate lower likelihood of depression relative to the reference group. Results with p < 0.05 are considered statistically significant.*
