# Supplementary material for: The impact of loneliness on depression, mental health, and physical well-being
Source: PLoS One. 2025 Jul 9;20(7):e0319311. doi: 10.1371/journal.pone.0319311 (PMC12240311; doi:10.1371/journal.pone.0319311)
Supplement: S3 Table — (DOCX) [file pone.0319311.s003.docx]

Supplementary Table S3: Racial and Ethnic Differences in the Association Between Loneliness and Number of Poor Mental Health Days (Marginal Effects Model)

|  | Race & Ethnicity | Margin | Std. Err. | t | 95% CI | | P>t |
| --- | --- | --- | --- | --- | --- | --- | --- |
| Lonely | Mental Health (Days) |  |  |  |  |  |  |
| Never | Black vs. White | -0.35 | 0.727 | -0.48 | -1.77 | 1.08 | 0.633 |
|  | Hispanic vs. White | -0.43 | 0.861 | -0.5 | -2.12 | 1.26 | 0.619 |
| Always | Black vs. White | -3.75 | 1.327 | -2.82 | -6.35 | -1.15 | **0.005** |
|  | Hispanic vs. White | -1.71 | 1.256 | -1.36 | -4.17 | 0.75 | **0.174** |
| Usually | Black vs. White | 0.49 | 1.428 | 0.35 | -2.3 | 3.29 | 0.729 |
|  | Hispanic vs. White | -0.83 | 1.378 | -0.6 | -3.53 | 1.87 | 0.546 |
| Sometimes | Black vs. White | -0.24 | 0.573 | -0.41 | -1.36 | 0.89 | 0.682 |
|  | Hispanic vs. White | 0.42 | 0.645 | 0.64 | -0.85 | 1.68 | 0.52 |
| Rarely | Black vs. White | -0.07 | 0.473 | -0.16 | -1 | 0.85 | 0.876 |
|  | Hispanic vs. White | -0.48 | 0.63 | -0.75 | -1.71 | 0.76 | 0.451 |

*Table 8 presents the marginal effects comparing Black and Hispanic individuals to White individuals in the association between loneliness and the number of poor mental health days, assessing race and ethnicity as moderators. Estimates represent differences in predicted number of poor mental health days across loneliness categories by racial/ethnic group. Models were adjusted for age, sex, marital status, employment, education, language, metro status, and included state, year, and month fixed effects. Only the "Always Lonely" category showed a statistically significant difference between Black and White individuals (p = 0.005).*
