# Supplementary material for: The impact of loneliness on depression, mental health, and physical well-being
Source: PLoS One. 2025 Jul 9;20(7):e0319311. doi: 10.1371/journal.pone.0319311 (PMC12240311; doi:10.1371/journal.pone.0319311)
Supplement: S4 Table — (DOCX) [file pone.0319311.s004.docx]

Supplementary Table S4: Age Differences in the Association Between Loneliness and Number of Poor Mental Health Days

|  | Age group | Margin | Std. Err. | t | 95% CI | | P>t |
| --- | --- | --- | --- | --- | --- | --- | --- |
| Lonely | Menth |  |  |  |  |  |  |
| Never | 45-64Yr. vs. 18-44Yr. | 1.32 | 0.413 | 3.18 | 0.51 | 2.13 | **<0.001** |
|  | >64Yr. vs. 18-44Yr. | 0.11 | 0.477 | 0.24 | -0.82 | 1.05 | 0.814 |
| Always | 45-64Yr. vs. 18-44Yr. | 0.17 | 0.703 | 0.25 | -1.2 | 1.55 | 0.805 |
|  | >64Yr. vs. 18-44Yr. | -1.66 | 1.262 | -1.47 | -3.87 | 0.55 | 0.141 |
| Usually | 45-64Yr. vs. 18-44Yr. | -0.77 | 0.728 | -1.05 | -2.2 | 0.66 | 0.292 |
|  | >64Yr. vs. 18-44Yr. | -0.88 | 0.908 | -0.97 | -2.66 | 0.9 | 0.332 |
| Sometimes | 45-64Yr. vs. 18-44Yr. | -0.43 | 0.31 | -1.4 | -1.04 | 0.17 | 0.161 |
|  | >64Yr. vs. 18-44Yr. | -2.15 | 0.408 | -5.26 |  | -4.3 | **<0.001** |
| Rarely | 45-64Yr. vs. 18-44Yr. | -0.22 | 0.24 | -0.91 | -0.69 | 0.25 | 0.36 |
|  | >64Yr. vs. 18-44Yr. | -1.03 | 0.39 | -2.63 |  | -2.06 | **0.009** |

*Table presents the marginal effects comparing middle-aged adults (45–64 years) and older adults (>64 years) to younger adults (18–44 years) in the association between loneliness and the number of poor mental health days. Estimates reflect differences in predicted number of poor mental health days across loneliness categories by age group. Models were adjusted for race/ethnicity, sex, marital status, employment, education, language, and metro status, with fixed effects for state, year, and month. Significant differences were observed primarily among adults gaged >64 years reporting "Sometimes" and "Rarely" feeling lonely (p < 0.001).*
