# Supplementary material for: The impact of loneliness on depression, mental health, and physical well-being
Source: PLoS One. 2025 Jul 9;20(7):e0319311. doi: 10.1371/journal.pone.0319311 (PMC12240311; doi:10.1371/journal.pone.0319311)
Supplement: S5 Table — (DOCX) [file pone.0319311.s005.docx]

Supplementary Table S5: Racial and Ethnic Differences in the Association Between Loneliness and Number of Poor Physical Health Days

|  | Age group | Margin | Std. Err. | t | 95% CI | | P>t |
| --- | --- | --- | --- | --- | --- | --- | --- |
| Lonely | Physical Health (Days) |  |  |  |  |  |  |
| Never | Black vs. White | -0.88 | 0.724 | -1.21 | -2.3 | 0.54 | 0.225 |
|  | Hispanic vs. White | -0.7 | 0.966 | -0.72 | -2.59 | 1.2 | 0.47 |
| Always | Black vs. White | -3.59 | 1.46 | -2.46 |  | -7.19 | **0.014** |
|  | Hispanic vs. White | -1.17 | 1.282 | -0.92 | -3.69 | 1.34 | 0.36 |
| Usually | Black vs. White | -0.31 | 1.52 | -0.2 | -3.3 | 2.67 | 0.838 |
|  | Hispanic vs. White | 0.52 | 1.425 | 0.37 | -2.27 | 3.31 | 0.714 |
| Sometimes | Black vs. White | 1.03 | 0.717 | 1.44 | -0.37 | 2.44 | 0.15 |
|  | Hispanic vs. White | 1.41 | 0.689 | 2.05 | 0.06 | 2.76 | **0.041** |
| Rarely | Black vs. White | 0.33 | 0.558 | 0.59 | -0.76 | 1.42 | 0.054 |
|  | Hispanic vs. White | 0.04 | 0.68 | 0.06 | -1.3 | 1.37 | 0.956 |

*Table 11 presents the marginal effects comparing Black and Hispanic individuals to White individuals in the association between loneliness and the number of poor physical health days, assessing race and ethnicity as moderators. Estimates reflect differences in predicted number of poor physical health days across loneliness categories by racial/ethnic group. Models were adjusted for age, sex, marital status, employment status, education level, language, and metro status, and included state, year, and month fixed effects. A statistically significant difference was observed for Black individuals in the "Always Lonely" category (p = 0.014) and for Hispanic individuals in the "Sometimes Lonely" category (p = 0.041).*
