# Supplementary material for: The impact of loneliness on depression, mental health, and physical well-being
Source: PLoS One. 2025 Jul 9;20(7):e0319311. doi: 10.1371/journal.pone.0319311 (PMC12240311; doi:10.1371/journal.pone.0319311)
Supplement: S6 Table — (DOCX) [file pone.0319311.s006.docx]

Supplementary Table S6: Age Differences in the Association Between Loneliness and Number of Poor Physical Health Days

|  | Age group | Margin | Std. Err. | t | 95% CI | | P>t |
| --- | --- | --- | --- | --- | --- | --- | --- |
| Lonely | Physical Health (Days) |  |  |  |  |  |  |
| Never | 45-64Yr. vs. 18-44Yr. | 4.07 | 0.59 | 6.9 | 2.92 | 5.23 | <0.001 |
|  | >64Yr. vs. 18-44Yr. | 3.85 | 0.71 | 5.4 | 2.46 | 5.25 | <0.001 |
| Always | 45-64Yr. vs. 18-44Yr. | 3.8 | 0.906 | 4.19 | 2.02 | 5.57 | <0.001 |
|  | >64Yr. vs. 18-44Yr. | 4.25 | 1.417 | 3 | 1.48 | 7.03 | 0.003 |
| Usually | 45-64Yr. vs. 18-44Yr. | 4.27 | 0.915 | 4.66 | 2.47 | 6.06 | <0.001 |
|  | >64Yr. vs. 18-44Yr. | 5.72 | 1.094 | 5.23 | 3.57 | 7.86 | <0.001 |
| Sometimes | 45-64Yr. vs. 18-44Yr. | 2.82 | 0.448 | 6.31 | 1.95 | 3.7 | <0.001 |
|  | >64Yr. vs. 18-44Yr. | 3.14 | 0.596 | 5.27 | 1.97 | 4.31 | <0.001 |
| Rarely | 45-64Yr. vs. 18-44Yr. | 3.27 | 0.437 | 7.49 | 2.41 | 4.13 | <0.001 |
|  | >64Yr. vs. 18-44Yr. | 3.53 | 0.606 | 5.82 | 2.34 | 4.72 | <0.001 |

*Table 12 presents the marginal effects comparing middle-aged adults (45–64 years) and older adults (>64 years) to younger adults (18–44 years) in the association between loneliness and the number of poor physical health days. Estimates reflect differences in predicted number of poor physical health days across loneliness categories by age group. Models were adjusted for race/ethnicity, sex, marital status, employment status, education level, language, and metro status, and included state, year, and month fixed effects. All differences were statistically significant (p < 0.001), indicating that both middle-aged and older adults consistently reported more poor physical health days across all loneliness categories compared to younger adults*.
