# Supplementary material for: The impact of loneliness on depression, mental health, and physical well-being
Source: PLoS One. 2025 Jul 9;20(7):e0319311. doi: 10.1371/journal.pone.0319311 (PMC12240311; doi:10.1371/journal.pone.0319311)
Supplement: Attachment — Submitted filename: Response to Reviewers.docx [file pone.0319311.s008.docx]

Response to Academic Editor

Thank you for your thoughtful feedback. We appreciate the opportunity to clarify key methodological aspects of our study.

Academic Editor:

*Comment :*

*This study investigates the associations of loneliness with depression, mental health, and physical health, accounting for confounders and state and year fixed effects. Data analysis used Inverse Probability Weighting (IPW) to estimate the Average Treatment Effect (ATE) of loneliness on depression, mental health days, and physical health days. Findings suggest loneliness as a strong predictor of depression and poor mental and physical health. While this method was used to minimize selection bias, please include some rationales underlying the use of matched and unmatched groups in the study methods.*

*Per the RECORD checklist, please provide information on the data cleaning methods used in the study.*

Rationale for Use of Matched and Unmatched Groups in the Study Methods

In this study, we applied Inverse Probability Weighting (IPW) rather than traditional propensity score matching. The rationale for using IPW lies in its ability to retain the full analytic sample (unmatched approach) while minimizing selection bias due to observed confounders. Unlike matching methods, which exclude unmatched individuals and potentially reduce generalizability, IPW allows for adjustment across all levels of loneliness by weighting individuals inversely proportional to their probability of being in a given loneliness category.

This approach was particularly suited to our objective of estimating the Average Treatment Effect (ATE) across a multi-level exposure (five categories of loneliness) within a nationally representative dataset. The use of IPW, combined with BRFSS sampling weights and state/year fixed effects, enabled us to balance covariates across groups without sacrificing sample size or representativeness.

We have clarified this rationale in the Statistical Analysis section of the manuscript to explicitly distinguish why a weighting strategy was preferred over matching techniques.

Data Cleaning Methods (RECORD Checklist Compliance)

In adherence to the RECORD guidelines, we have expanded the Methodology section to detail our data cleaning procedures:

We conducted initial data integrity checks to identify incomplete or inconsistent responses.

Participants were excluded if they provided responses such as "Don’t know," "Refused," or had missing data for key variables, including the exposure (loneliness), outcomes (depression diagnosis, poor mental health days, poor physical health days), and covariates.

We verified coding consistency across survey years (2016–2023) to ensure harmonization of variable definitions.

Extreme values for continuous variables (e.g., health days) were assessed, and implausible entries outside the BRFSS-defined ranges were removed.

For the IPW procedure, we trimmed extreme weights at the 1st and 99th percentiles to mitigate undue influence from outliers.

All cleaning steps were performed using Stata, with code documentation available upon request to ensure transparency and reproducibility.

We have updated the manuscript to explicitly describe these steps under a new subsection titled Data Cleaning and Preparation within the Methodology section.

We trust that these clarifications address your concerns. We remain committed to ensuring methodological transparency and adherence to best practices.

Response to Reviewers

We are grateful for the thoughtful and constructive feedback provided by both reviewers. We have carefully considered each comment and revised the manuscript accordingly to strengthen clarity, methodological transparency, and overall contribution. Below we provide a point-by-point response detailing how each comment has been addressed.

Reviewer #1

*Comment: “The introduction effectively outlines the importance of studying loneliness and clearly defines the key health outcomes of interest. Please expand on the limitations of previous studies and how this study advances current knowledge. What gaps in the literature does this work specifically address?”*

Response:

We appreciate this suggestion and have revised the introduction to explicitly highlight the limitations of previous research, including over-reliance on correlational designs, use of non-representative samples, and insufficient attention to the interplay between loneliness and social determinants of health. We now clarify how our study addresses these gaps through the use of a large, nationally representative dataset (BRFSS), application of inverse probability weighting to approximate causal inference, and exploration of demographic variations. These revisions can be found in the revised Introduction section.

*Comment: “You note that social determinants may influence the relationship between loneliness and health outcomes, yet they are treated solely as covariates rather than moderators. Although subgroup analyses for race and ethnicity are included, a more granular exploration of how specific socioeconomic and structural factors contribute to disparities could provide additional insights.”*

Response:

We agree with the reviewer that moderator analysis offers further insight into disparities. While our initial analyses treated social determinants as covariates, we have added subgroup analyses stratified by sex, race/ethnicity, and age groups.

*Comment: “Regarding the methods, please describe the inclusion and exclusion criteria for BRFSS participants.”*

Response:

We have revised the Methodology section to clearly define the inclusion and exclusion criteria. Participants were included if they were aged 18 or older and had complete responses to the loneliness measure, all three outcome variables, and all covariates. Participants who responded “Don’t know,” “Refused,” or had missing data were excluded.

*Comment: “Clarify how the survey design ensures national representativeness.”*

Response:

We have added a paragraph explaining the BRFSS's complex multistage sampling design and the application of sampling weights to ensure state and national representativeness. These revisions are now detailed in the Methodology section.

*Comment: “Further detail on how covariates were coded (e.g., categorical vs. continuous) would strengthen transparency.”*

Response:

We have expanded the Covariates and Coding subsection to clearly indicate how each variable was treated in the analysis (e.g., age as continuous, education as categorical with dummy coding, etc.).

*Comment: “Please provide stepwise equations for IPW estimation, including the propensity score model, how inverse probabilities were computed, and the final weighting approach.”*

Response:

We have added a detailed, step-by-step explanation of the IPW process in the Statistical Analysis section, including the multinomial logistic regression used to generate propensity scores, computation of inverse probabilities, trimming of extreme weights at the 1st and 99th percentiles, and the final multiplication with BRFSS sampling weights.

*Comment: “How were sampling weights from BRFSS incorporated into the IPW procedure? Were they applied multiplicatively? Did you implement strategies to address extreme propensity scores?”*

Response:

Yes, the BRFSS sampling weight (pweight = _llcpwt) was applied multiplicatively with the IPW weights to create the final analysis weight. We have now clarified this process in the methodology and included code examples. We also describe our trimming of extreme weights at the 1st and 99th percentiles to reduce their influence.

Comment: *“Additional details on how the ATE was computed and what the reported values represent in the context of loneliness and health outcomes would be helpful.”*

Response:

We expanded the Interpretation of ATE Estimates subsection to explain how the ATE represents the average differences in outcomes between loneliness levels, adjusted for confounders. We provide clear examples (e.g., “10.8 more poor mental health days for ‘Always lonely’ vs. ‘Never lonely’ individuals”).

*Comment: “Would you be open to providing the statistical code?”*

Response:

Yes. We have stated in the revised manuscript that the full annotated Stata code is available upon request and will also be posted on a public GitHub repository upon acceptance for publication. This is now noted in the Software and Reproducibility section.

*Comment: “The discussion effectively contextualizes the findings, but the contribution relative to prior studies could be made more explicit... Strengthening the discussion on mechanisms and policy implications would further enhance its contribution.”*

Response:

We revised the Discussion to clearly articulate how our study builds on prior work—emphasizing the use of causal methods, nationally representative data, and the joint analysis of mental and physical health outcomes. We also elaborated on the biological and psychosocial mechanisms linking loneliness to health outcomes and expanded the section on policy and clinical implications to include practical interventions (e.g., social prescribing, digital tools, community investments).

Reviewer #2

Comment: *“How was self-diagnosis avoided regarding depression? Was there a mechanism used to ensure that data collected regarding diagnosis is accurate?”*

Response:

We appreciate this important question. The BRFSS depression item specifically asks whether a healthcare professional has ever told the respondent they had depression. This helps mitigate concerns about self-diagnosis and aligns the variable more closely with clinical depression. We have clarified this in the Discussion section under “Addressing Self-Reported Limitations.”

Once again, we thank both reviewers for their insightful comments, which have significantly improved the clarity and impact of the manuscript. We believe these revisions address all concerns and enhance the scientific rigor, transparency, and contribution of our work.
